# Laparoscopic Ligation of the Inferior Mesenteric Artery: A Systematic Review of an Emerging Trend for Addressing Type II Endoleak Following Endovascular Aortic Aneurysm Repair

**DOI:** 10.3390/jcm13092584

**Published:** 2024-04-27

**Authors:** Konstantinos Roditis, Paraskevi Tsiantoula, Nikolaos-Nektarios Giannakopoulos, Afroditi Antoniou, Vasileios Papaioannou, Sofia Tzamtzidou, Dimitra Manou, Konstantinos G. Seretis, Theofanis T. Papas, Nikolaos Bessias

**Affiliations:** Department of Vascular Surgery, Korgialenio-Benakio Hellenic Red Cross Hospital, 115 26 Athens, Greece; tsiantoulapar@0310.syzefxis.gov.gr (P.T.); giannakopoulosnik@0310.syzefxis.gov.gr (N.-N.G.); antoniouafr@0310.syzefxis.gov.gr (A.A.); papaioannouvas@0310.syzefxis.gov.gr (V.P.); tzamtzidousof@0310.syzefxis.gov.gr (S.T.); manoudim@0310.syzefxis.gov.gr (D.M.); seretiskon@0310.syzefxis.gov.gr (K.G.S.); papastheo@0310.syzefxis.gov.gr (T.T.P.); besiasnik@0310.syzefxis.gov.gr (N.B.)

**Keywords:** abdominal aortic aneurysm, endoleak, inferior mesenteric artery, laparoscopic surgical procedure, endovascular aneurysm repair, ligation, postoperative complication, reoperation

## Abstract

**Background/Objectives**: this systematic review aims to explore the efficacy and safety of the laparoscopic ligation of the inferior mesenteric artery (IMA) as an emerging trend for addressing a type II endoleak following endovascular aortic aneurysm repair (EVAR). **Methods**: A comprehensive literature search was conducted across several databases including Medline, Scopus, and the Cochrane Central Register of Controlled Trials, adhering to the PRISMA guidelines. The search focused on articles reporting on the laparoscopic ligation of the IMA for the treatment of a type II endoleak post-EVAR. Data were extracted regarding study characteristics, patient demographics, technical success rates, postoperative outcomes, and follow-up results. **Results**: Our analysis included ten case studies and two retrospective cohort studies, comprising a total of 26 patients who underwent a laparoscopic ligation of the IMA between 2000 and 2023. The mean age of the cohort was 72.3 years, with a male predominance (92.3%). The mean AAA diameter at the time of intervention was 69.7 mm. The technique demonstrated a high technical success rate of 92.3%, with a mean procedure time of 118.4 min and minimal blood loss. The average follow-up duration was 19.9 months, with 73% of patients experiencing regression of the aneurysmal sac, and no reports of an IMA-related type II endoleak during the follow-up period. **Conclusions**: The laparoscopic ligation of the IMA for a type II endoleak following EVAR presents a promising, minimally invasive alternative with high technical success rates and favorable postoperative outcomes. Despite its potential advantages, including reduced contrast agent use and radiation exposure, its application remains limited to specialized centers. The findings suggest the need for further research in larger prospective studies to validate the effectiveness of this procedure and potentially broaden its clinical adoption.

## 1. Introduction

Improved outcomes in abdominal aortic aneurysm (AAA) management via endovascular aneurysm repair (EVAR) have been facilitated by advancements in endovascular techniques and devices, alongside the refinement of operators’ expertise [1]. Despite EVAR’s evolution as the preferred treatment option [2,3], it is important to acknowledge its potential association with adverse outcomes such as endoleaks and endograft occlusion. Consequently, there remains a notable need for subsequent interventions for patients undergoing EVAR, necessitating prolonged follow-up periods and lifelong surveillance by computed tomography angiography (CTA) [4], in contrast with open aortic aneurysm repair [2,3].

A type II endoleak is frequently detected post-EVAR [5,6] due to retrograde collateral circulation via open aortic branches such as the inferior mesenteric artery (IMA), lumbar arteries, or the median sacral artery [7]. Type II endoleaks are the most common type of endoleak, occurring and identified early after EVAR or at a later time during patient postoperative surveillance. A follow-up study by Lo et al. showed that in 2367 post-EVAR patients, 18% experienced early type II endoleaks that subsided soon after, 5% had persistent ones, and 11% acquired novel ones during follow-up [6]. This persistent flow, observed in 45% to 85% of all type II endoleaks [8,9,10], poses risks of aneurysm sac enlargement, necessitating reintervention or potentially leading to aortic ruptures [11,12]. The absence of a circumferential thrombus in the aneurysmal sac or large flow lumen, the number of patent aortic branches arising from the aneurysm, IMA patency, the number of patent lumbar arteries > 3, a diameter of the patent IMA ≥ 3 mm, a diameter of lumbar arteries ≥ 2 mm, and anticoagulation therapy have been consistently reported as risk factors associated with persistent or late-developing type II endoleaks after EVAR. On the other hand, coil embolization of internal iliac arteries, increasing age, female sex, the absence of chronic obstructive pulmonary disease (COPD), chronic renal disease (CRD), arterial hypertension, graft type, absence of post-implantation syndrome (PIS), no smoking history, and no peripheral arterial disease (PAD) history have been occasionally mentioned in the literature as risk factors for type II endoleak development, albeit without certainty [13]. About 50% of the patients with persistent or late-occurring type II endoleaks develop an aneurysm sac growth, with half of them needing a secondary intervention at 24 months. In a recently published meta-analysis which included 2643 patients with a type II endoleak deriving from 33 observational studies, 54% of them were identified in less than 30 days of follow-up and 8% after the first year. Those who were diagnosed early seemed to have the problem resolved more often in contrast with those diagnosed at a later point (OR 2.41). An aneurysmal sac growth due to the type II endoleak was reported in 29% and aneurysm rupture in 1.1% [14]. Although some type II endoleaks may spontaneously resolve, those persisting often require intervention. The management of type II endoleaks presents a complex challenge requiring a multifaceted approach that ranges from transarterial to surgical interventions [15]. The introduction of technical tips, such as those described by Touma et al., signifies the evolution of minimally invasive techniques to simplify the repair process [16]. 

Traditionally, the transarterial embolization of the IMA via the middle colic artery has been the go-to method [17,18]. However, recent studies have cast doubt on its efficacy, suggesting a high rate of treatment failure associated with this approach [19,20]. Other approaches to managing type II endoleaks, such as translumbar and transcaval embolization, are also available with varied efficacy rates and results [17,21]. Video laparoscopic surgery, offering potential advantages such as its suitability for patients with chronic renal insufficiency (due to the absence of contrast agents) and serving as an alternative in cases of unsuccessful endovascular interventions, involves clipping or ligating the origin of the inferior mesenteric artery (IMA). This technique also eliminates radiation exposure and may result in fewer artifacts in subsequent procedures. However, despite its proved efficiency in many surgical disciplines (i.e., general surgery, urology) and it being an emerging trend lately also in vascular surgery, given its benefits, its utilization remains limited to specialized centers, and widespread adoption has not yet occurred [22].

The present systematic review aimed at searching the existing literature for cases of the laparoscopic ligation of the IMA to treat a type II endoleak after EVAR, as well in assessing the midterm efficacy and safety of the procedure.

## 2. Materials and Methods

### 2.1. Literature Search and Study Selection Process

A thorough search of the literature was undertaken using the databases Medline (https://www.pubmed.gov, accessed on 20 March 2024), Scopus (https://www.scopus.com, accessed on 2 April 2024), and Cochrane Central Register of Controlled Trials (https://www.cochranelibrary.com/central, accessed on 2 April 2024). All studies reporting on laparoscopic ligation of the IMA for managing type II endoleak following EVAR were included in our review. We excluded studies published in languages other than English, those that referred to preventive IMA embolization prior to EVAR, and treatment of colon cancer. Manual search of the reference sections of initially identified articles was also undertaken to check for any further reported cases. The following Medical Subject Headings (MESH) search terms were combined in our search: “laparoscopic ligation”, “inferior mesenteric artery”, “IMA”, “type II endoleak”, “endovascular aneurysm repair”, and “EVAR”.

Initially located articles had their abstracts and titles independently screened by three authors (K.R., N.-N.G., and P.T.) and any disputes were resolved after consensus. Selected articles were subsequently examined to see whether they were suitable for inclusion, as well as to avoid the inclusion of duplicate cases.

### 2.2. Data Extraction

**Figure 1:**
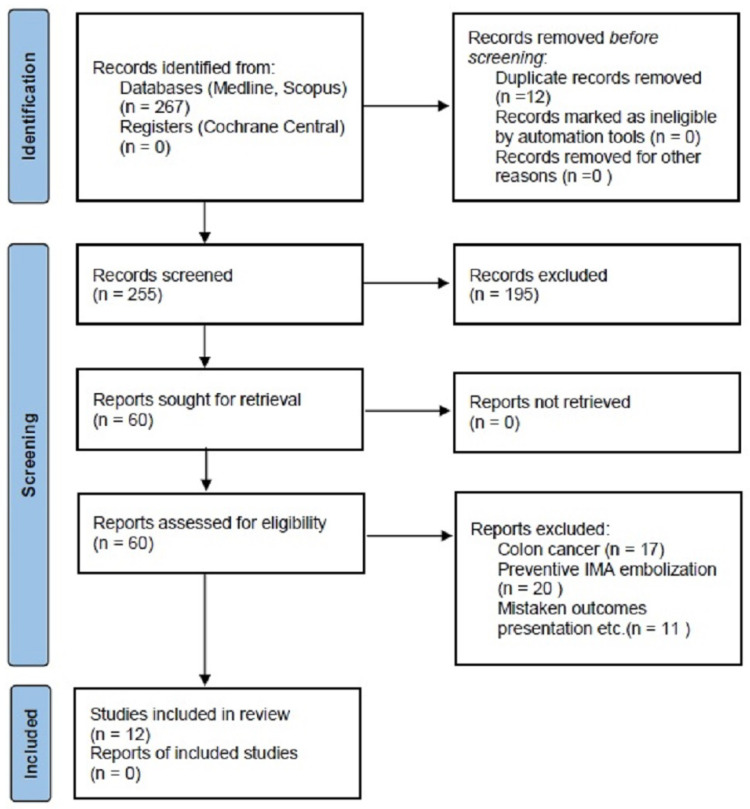
PRISMA flow chart of the study selection process. IMA = inferior mesenteric artery.

To streamline data collection, a standardized Excel 2019 (Microsoft, Redmond, WA, USA) spreadsheet was created for organizing and analyzing data exclusively from texts and tables of selected studies, without seeking additional information from authors. Data extraction was conducted independently by three reviewers (K.R., N.-N.G., and P.T.) and any disputes between reviewers were resolved through discussion and consensus, with involvement of a third reviewer if necessary (T.T.P.). Extracted data encompassed study characteristics (journal type, publication date, study group variability, and treatment methods), participant demographics (age, sex, and mean abdominal aortic aneurysm (AAA) diameter), clinical and procedural details (intervention timing, average hospital stay, warfarin, and direct oral anticoagulant or any other anticoagulant administration). This review encompasses various parameters including comorbidities such as arterial hypertension, hyperlipidemia, diabetes mellitus, coronary artery disease, chronic kidney disease, obesity, and chronic obstructive pulmonary disease. Additionally, operative data (such as duration of operation, laparoscopic techniques, concurrent procedures, and blood loss), outcomes (including technical success rate, 30-day mortality, survival rates, and AAA diameter during follow-ups), and complications (like open conversion, persistent type II endoleak, and re-intervention) were also considered. The review protocol, including selection and reporting processes, followed the 2020 Preferred Reporting Items for Systematic Reviews and Meta-Analyses (PRISMA) guidelines [23] (Figure 1).

### 2.3. Statistical Analysis

We report only descriptive data, with our systematic review not being comparative; therefore, no statistical analysis of presented data was performed, whatsoever.

## 3. Results

Our research included a detailed examination of ten case studies and two retrospective cohort studies covering, from 2000 to 2023, a total of 26 patients who underwent a laparoscopic ligation of the inferior mesenteric artery (IMA) following endovascular aneurysm repair (EVAR) to address a persistent type II endoleak [24,25,26,27,28,29,30,31,32,33,34,35]. The patients exhibited a mean age of 72.3 ± 7.6 years, with a predominance of males, constituting 92.3% (24 out of 26). The mean diameter of the abdominal aortic aneurysm (AAA) at the time of the video laparoscopic surgery was recorded at 69.7 ± 20.3 mm. The demographic data and comorbidities of the patients are delineated in Table 1. The duration between the initial endovascular repair and the subsequent intervention ranged from 6 to 36 months, with the majority of patients remaining asymptomatic, except for one individual who presented with abdominal pain and necessitated emergent surgical intervention. Among the patient cohort, 15 individuals demonstrated an enlargement of the aneurysm sac compared to the preprocedural AAA diameter, either by more than 5 mm within 6 months or by more than 1 cm from the preoperative diameter. Furthermore, in 11 patients, despite the stability of the sac diameter, the continued presence of the endoleak necessitated therapeutic intervention, as determined by the attending physician.

No correlation was seen between the continuous use of antiplatelet or anticoagulant therapy and sac enlargement or failure to shrink, as well as the persistence of a type II endoleak in the cohort of patients included in our analysis. 

### 3.1. Surgical Technique and 30-Day Outcomes

Generally, video laparoscopic ligation was chosen for patients without a hostile abdomen, those showing no signs of an inflammatory AAA, or those with significant cardiac risk factors. The laparoscopic ligation of the IMA was performed through a transperitoneal approach under general anesthesia in all instances. Typically, the procedure involved the insertion of three trocars, usually consisting of one 10 mm trocar and two 5 mm trocars. Pneumoperitoneum was achieved by the open insertion of a blunt 10 mm trocar at the umbilical level. Additional ports were positioned in the xiphoid process and in the right iliac fossa. In two cases, the da Vinci robotic system from Intuitive Surgical, based in Sunnyvale, California, was utilized. The dissection of the IMA from its origin at the aneurysmal sac was performed in all cases, with nonabsorbable clips utilized to secure its ligation, without harming the neighboring hypogastric plexus. Additional procedures were performed in five patients, including the simultaneous ligation of the right internal iliac artery, retroperitoneal access and the endoscopic clipping of a patent lumbar artery at the L5 level, the ligation of lumbar arteries, plus a thrombin injection in the aneurysmal sac. In one patient, the laparoscopic ligation of the IMA was performed after transarterial coil embolization had previously failed [33]. The mean duration of the procedures was 118.4 ± 63.9 min, with minimal blood loss and no requirement for concentrated red blood cell transfusions. The intraoperative confirmation of IMA ligation was achieved using digital subtraction angiography (DSA), while postoperative confirmation was conducted through duplex ultrasound, contrast-enhanced ultrasound (CEUS), or computed tomography angiography (CTA), as outlined in Table 2. The technical success rate was 92.3% (24 out of 26 cases), with two initially unsuccessful cases undergoing reoperation within 24 h, resulting in successful laparoscopic revisions. The main cause for reoperation was either a patent branch of the inferior mesenteric artery (IMA) or a set of patent lumbar arteries that contributed to the type II endoleak, which had not been detected during the initial procedure. The mean hospital stay was 2.7 ± 0.7 days, with no perioperative or 30-day mortality recorded among the 26 patients. Furthermore, no instances of colonic ischemia were reported, and none of the patients required conversion to an open procedure.

### 3.2. Follow-Up Outcomes

The average follow-up duration was 19.9 ± 11.1 months. Among the patients, 73% (19 out of 26) experienced the regression of the aneurysmal sac compared to preoperative measurements, while in the remaining patients, the sac diameter remained stable. Throughout the follow-up period, seven out of the fifteen patients initially treated for aneurysmal sac enlargement exhibited sac regression, four maintained a stable sac diameter, and there were no recorded data for the remaining four. In these cases, there were no occurrences of open conversion or endovascular reintervention for a type II endoleak. Additionally, during the follow-up period, there were no instances of IMA-related type II endoleaks detected.

## 4. Discussion

The management of type II endoleaks remains a contentious issue, largely due to an incomplete understanding of their natural history. Studies by Jones et al. [36] and Wyss et al. [37] have highlighted the association between persistent type II endoleaks and adverse outcomes, including aneurysmal sac growth and the need for conversion to open repair or reintervention [31,32]. Conversely, Sidloff et al. reported a low incidence of rupture secondary to the isolated type II endoleaks, emphasizing the rarity of this complication [38]. However, it is important to note that a significant number of rupture cases post-EVAR take place even without the expansion of the sac. These findings underscore the complex nature of type II endoleaks and the challenges in their management. The critical role of post-EVAR surveillance in detecting and managing Type II endoleaks cannot be overstated, with contrast-enhanced ultrasound emerging as a pivotal tool for patients with renal insufficiency. Moreover, the advent of endovascular devices such as the Nellix endograft system represents a forward leap in our quest to mitigate the incidence of Type II endoleaks [15].

Open surgical repair with the sacotomy and suturing of the feeding vessels is a well-described and traditionally used technique, appearing to be associated with better results regarding the exclusion of the aneurysm, albeit with more serious complications, when compared to endovascular embolization [39]. Moulakakis et al. underscore the special attention that needs to be paid during open surgical maneuvers for ligating the culprit artery or arteries, as this may lead to possible catastrophic bleeding and even necessitate endograft explantation [39]. On the other hand, Akmal et al. have recently argued that despite the multifaceted endovascular options for treating persistent type II endoleaks after an initially successful EVAR, currently, available peer-reviewed data on the long-term follow-up of patients undergoing transarterial embolization techniques to address the problem do not provide sufficient evidence for national and international guidelines to recommend the best treatment choice [40].

While translumbar and transcaval embolization techniques have demonstrated high success rates with minimal complications [17,21], a laparoscopic intervention for type II endoleaks has not gained widespread acceptance. One possible explanation for its limited adoption is the requirement for specialized training, as well as the need for collaboration between vascular and general surgeons [27]. However, research indicates that the laparoscopic repair of aortic aneurysms is both feasible and safe, even in elective settings, suggesting a potential role for this technique in select cases [41]. Further advancements in laparoscopic techniques have resulted in the development of more sophisticated procedures, including laparoscopic sac fenestration, aimed at addressing post-implantation aneurysm growth [42]. In light of the technical challenges associated with traditional approaches, the technique described by Touma et al. offers a simplified and effective alternative for laparoscopic repair, underscoring the importance of evolving our strategies to improve patient outcomes [16].

Current recommendations in clinical practice suggest taking action for patients with type II endoleaks when the sac diameter surpasses a specific threshold in subsequent evaluations [4,13]. Nevertheless, other approaches for interventions at earlier stages of growth have been suggested, especially in instances of continuous endoleaks without sac enlargement. [43]. Early changes in the sac diameter have been identified as strong predictors of late outcomes after EVAR, emphasizing the importance of timely intervention, while major sac shrinkage is a good outcomes predictor for at least 5 years postoperatively [44]. The incidence of IMA-related type II endoleaks may be predictable based on preoperative assessments and studies which have shown an association between a larger cross-sectional area of the aortic lumen at the level of the IMA ostium and the development of type II endoleaks post-EVAR [9]. The preoperative embolization of the IMA has been suggested as a potential preventive measure against the development of type II endoleaks and subsequent interventions [10,45,46].

Despite the promising outcomes observed in our study regarding laparoscopic ligation for IMA-related type II endoleaks, several limitations should be acknowledged. The quality of the available data is relatively low, primarily consisting of case reports and only two case cohort studies. Additionally, the procedures were performed in specialized centers with significant laparoscopic expertise, limiting the generalizability of the results to broader clinical settings. Future research focusing on larger prospective studies is warranted to further elucidate the optimal management strategies for type II endoleaks and improve patient outcomes.

## 5. Conclusions

Despite recent technological advancements in managing abdominal aortic aneurysms (AAA) through endovascular repair (EVAR), challenges do remain. Researchers and surgeons have focused particularly on type II endoleaks as a common post-EVAR complication. Despite the benefits of EVAR, type II endoleaks can lead to aneurysm sac growth and necessitate further interventions. The complexity of managing type II endoleaks is evident, thus the critical role of surveillance post-EVAR is of fundamental importance. Traditional approaches like transarterial embolization have shown efficacy concerns, prompting the exploration of the laparoscopic technique as a treatment option for type II endoleaks. This minimally invasive technique seems to demonstrate high success rates and zero 30-day mortality, while researchers worldwide are advocating for larger prospective studies to validate optimal treatment strategies. The laparoscopic ligation of the IMA is considered an alternative intervention for type II endoleaks after EVAR endovascular techniques have not been successful or are not feasible due to anatomical considerations. This approach is typically reserved for patients who are suitable candidates for laparoscopic surgery, including those without a hostile abdomen, inflammatory AAA, or significant risk factors that would preclude general anesthesia and laparoscopic procedures. Despite promising outcomes, the widespread adoption of laparoscopic ligation for type II endoleaks is limited by the specialized expertise required in laparoscopic techniques and the need for collaboration between vascular and general surgeons. The laparoscopic ligation of aortic side branches currently occupies a niche role in the management algorithm for type II endoleaks after EVAR, serving as an effective salvage therapy when other interventions have been unsuccessful or contraindicated. It is not typically considered as a first-line approach, but rather as a valuable option in specific patient scenarios and its role continues to be defined within the context of patient selection, procedural success rates, and ongoing advancements in minimally invasive surgical techniques.

## Figures and Tables

**Table 1:** Patients’ demographics and comorbidities.

Study (Year of Publication)	No. of Cases	Mean Age, Years *	AAA Diameter, mm	HT	HL	CAD	COPD	DM	CKD	Obesity
Sirignano (2022) [24]	3	77 ± 8	64.7 ± 14	2/3	1/3	1/3	1/3	0/3	0/3	NA
San Norberto et al. (2019) [25]	1	72	62	NA	NA	NA	NA	NA	NA	NA
Morelli et al. (2019) [26]	2	76 ± 5.7	72.5 ± 3.5	1/2	0/2	0/2	1/2	0/2	0/2	NA
Piffaretti et al. (2017) [27]	11	76 ± 10	60	9/11	NA	6/11	2/11	1/11	2/11	2/11
Zou et al. (2014) [28]	1	55	70	NA	NA	NA	NA	NA	NA	NA
Lin et al. (2009) [29]	1	84	61	1/1	NA	0/1	1/1	0/1	0/1	1/1
Feezor et al. (2006) [30]	1	72	67	0/1	1/1	0/1	0/1	0/1	0/1	0/1
Zhou et al. (2006) [31]	1	61	NA	NA	NA	NA	NA	NA	1/1	NA
Karkos et al. (2005) [32]	1	76	129	NA	NA	NA	NA	NA	NA	NA
Ho et al. (2004) [33]	1	74	60	1/1	1/1	0/1	0/1	0/1	0/1	0/1
Richardson et al. (2003) [34]	2	70.2	55	NA	NA	NA	NA	NA	NA	NA
Wisselink et al. (2000) [35]	1	74	65	NA	NA	NA	NA	NA	NA	NA
Total	26	72.3 ± 7.6	69.7 ± 20.3	14/19	3/7	7/19	5/19	1/19	3/20	3/14

* Means are provided with ± 1 standard deviation (when available), AAA = abdominal aortic aneurysm, HT = arterial hypertension, HL = hyperlipidemia, CAD = coronary artery disease, COPD = chronic obstructive pulmonary disease, DM = diabetes mellitus, CKD = chronic kidney disease, NA = not applicable.

**Table 2:** Peri-procedural details and outcome.

Study (Year of Publication)	Procedural duration, Min *	Red Blood Cell Units Transfused	Evaluation of Technical Success	Technical Success	Accompanying Procedures	Conversion to Open Surgery
Sirignano (2022) [24]	NA	NA	NA	3/3	0	0
San Norberto et al. (2019) [25]	132	0	Postoperative CTA	1/1	Thrombin injected into aneurysmal sac	0
Morelli et al. (2019) [26]	182.5 ± 3.5	0	Postoperative CEUS	2/2	Endoscopic clipping of a patent lumbar artery	0
Piffaretti et al. (2017) [27]	97 ± 46	0	Postoperative duplex scan	11/11	0	0
Zou et al. (2014) [28]	50	0	NA	1/1	Laparoscopic clipping of right IIA	0
Lin et al. (2009) [29]	249	0	NA	1/1	0	0
Feezor et al. (2006) [30]	NA	0	Immediate postoperative CTA	1/1	0	0
Zhou et al. (2006) [31]	NA	0	Intraoperative DSA	1/1	0	0
Karkos et al. (2005) [32]	80	0	Intraoperative DSA	1/1	1000 units of thrombin injected in the aneurysmal sac	0
Ho et al. (2004) [33]	60	0	Postoperative duplex scan	1/1	Previous failed coil embolization of IMA	0
Richardson et al. (2003) [34]	85	0	24 h postoperative CTA	2/2 (1 reoperation in 24 h)	NA	0
Wisselink et al. (2000) [35]	130	0	24 h postoperative CTA	1/1 (reoperation in 24 h)	Lumbar arteries clipping, and in reoperation, thrombin was injected in the aneurysmal sac	0
	118.4 ± 63.9	0/25		24/26 **		0/26

* Duration in min is provided ± 1 standard deviation (where available). CTA = Computed tomography angiography, CEUS = contrast-enhanced ultrasound, DSA = digital subtraction angiography, IIA = internal iliac artery, IMA = inferior mesenteric artery, NA = not applicable. ** 26/26 following reoperation 24 h after initial procedure.

## Data Availability

The raw data supporting the conclusions of this article will be made available by the correspondence author on request.
